# Supplementary figures and images for: Direct Intracellular Visualization of Ebola Virus-Receptor Interaction by In Situ Proximity Ligation
Source: mBio. 2021 Jan 12;12(1):e03100-20. doi: 10.1128/mBio.03100-20 (PMC7844541; doi:10.1128/mBio.03100-20)

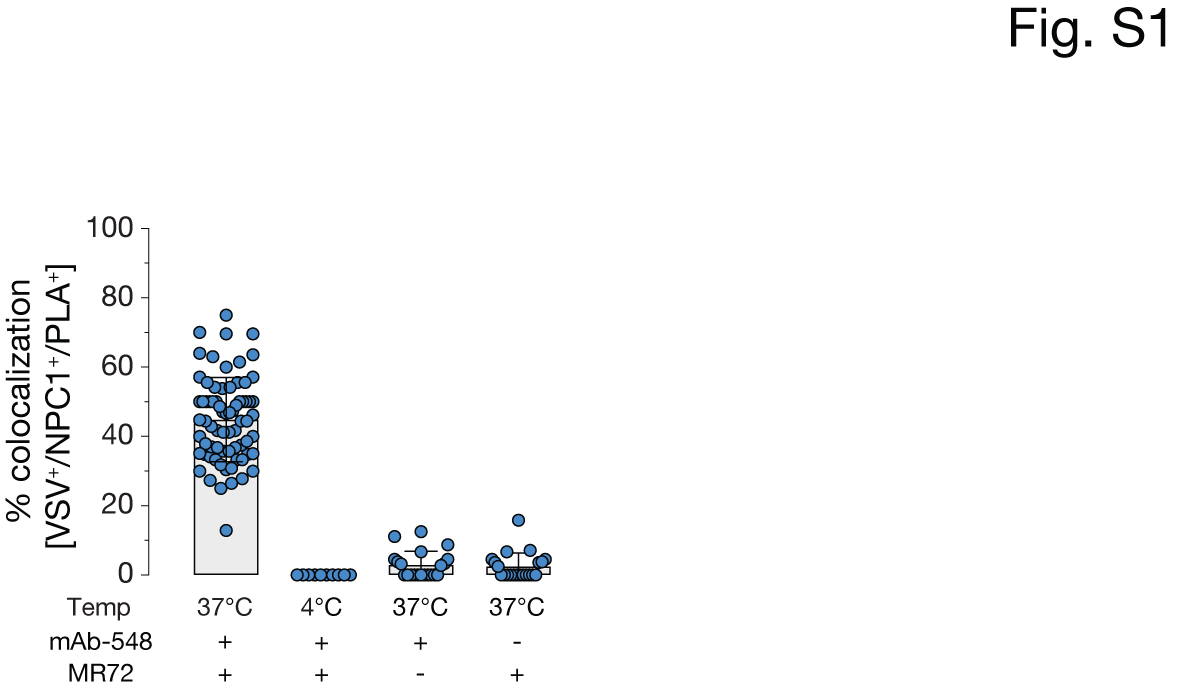

Supplement: FIG S1 [file mBio.03100-20-sf001.tif]

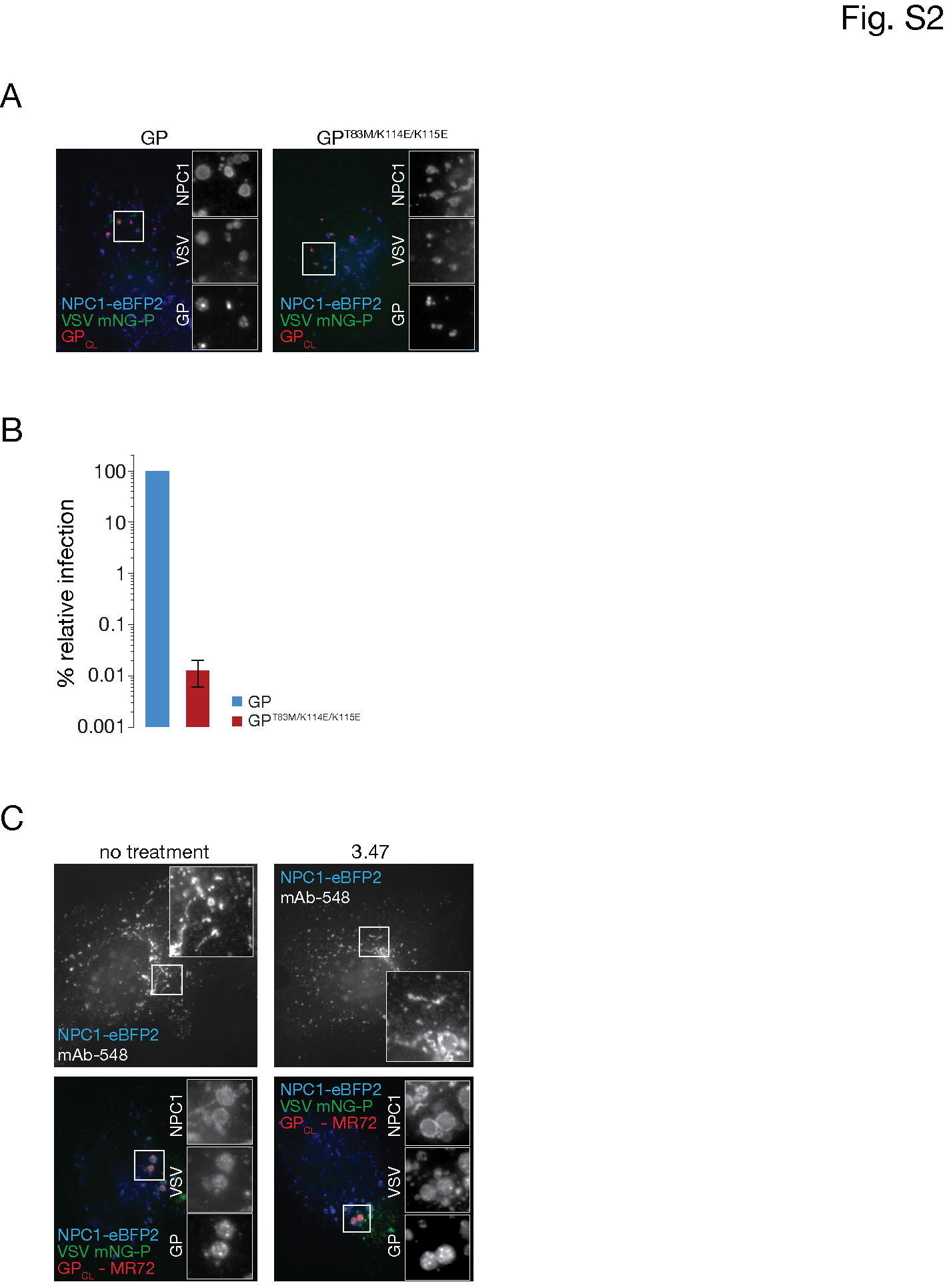

Supplement: FIG S2 [file mBio.03100-20-sf002.tif]

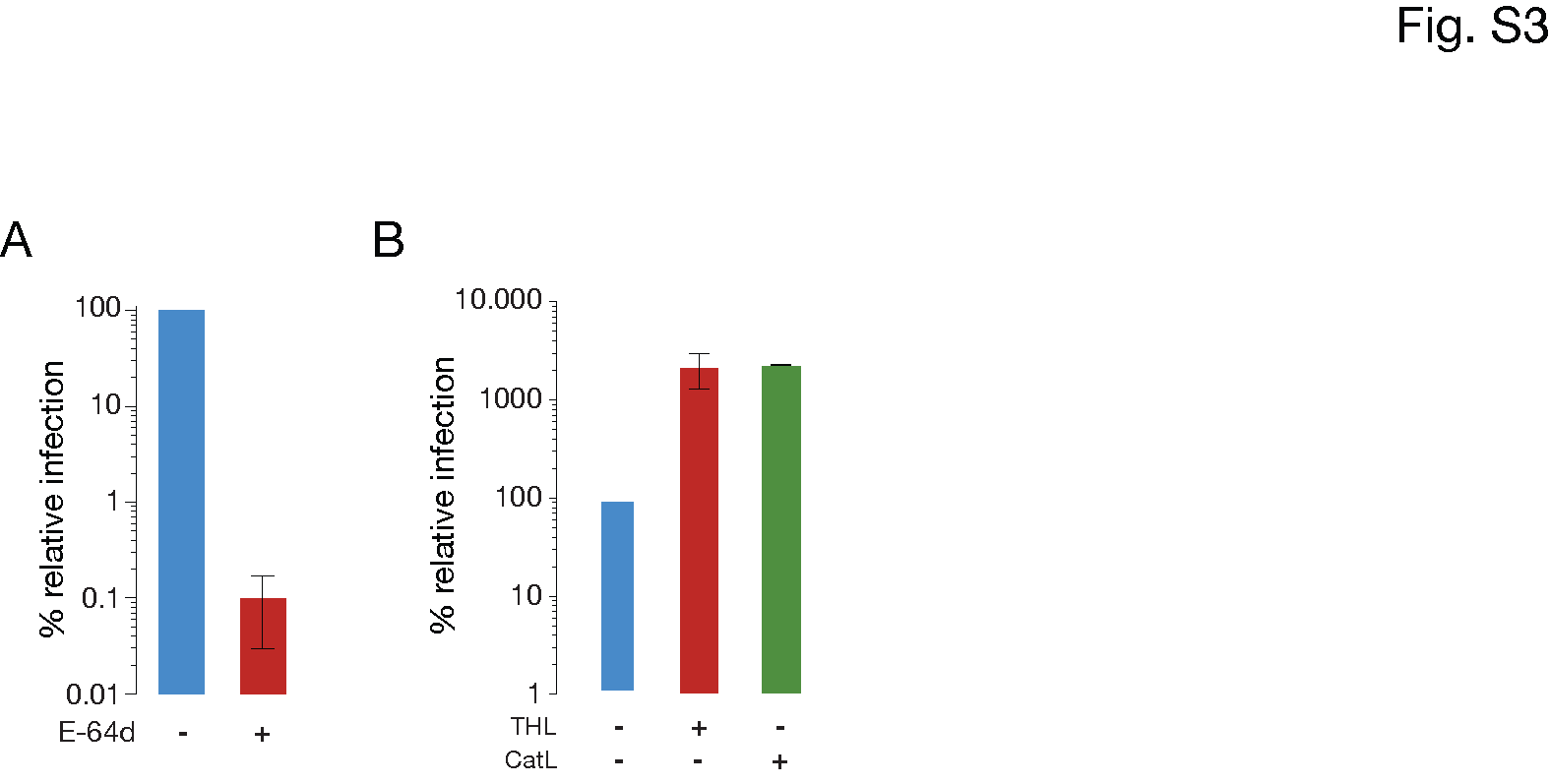

Supplement: FIG S3 [file mBio.03100-20-sf003.tif]

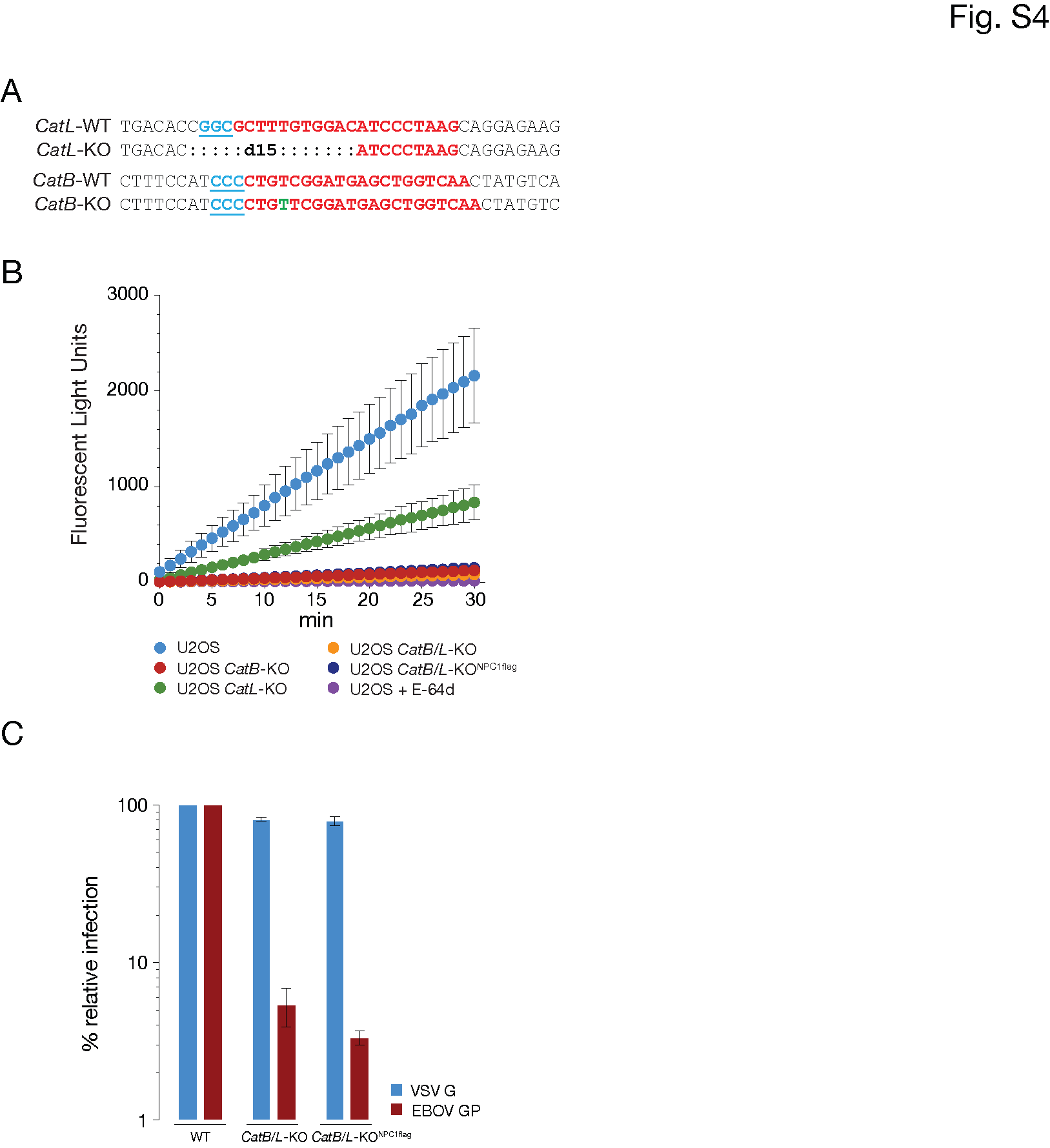

Supplement: FIG S4 [file mBio.03100-20-sf004.tif]

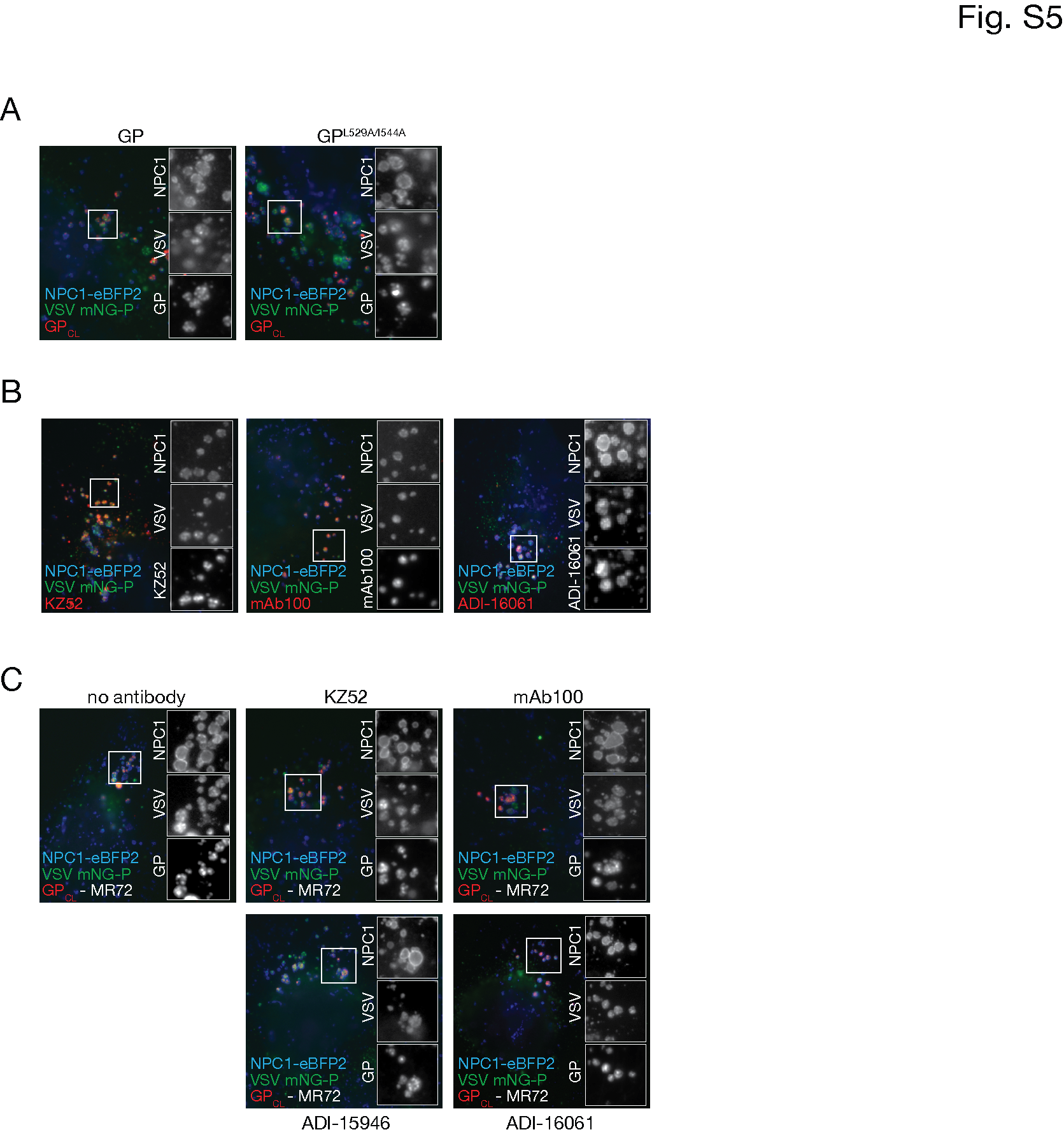

Supplement: FIG S5 [file mBio.03100-20-sf005.tif]

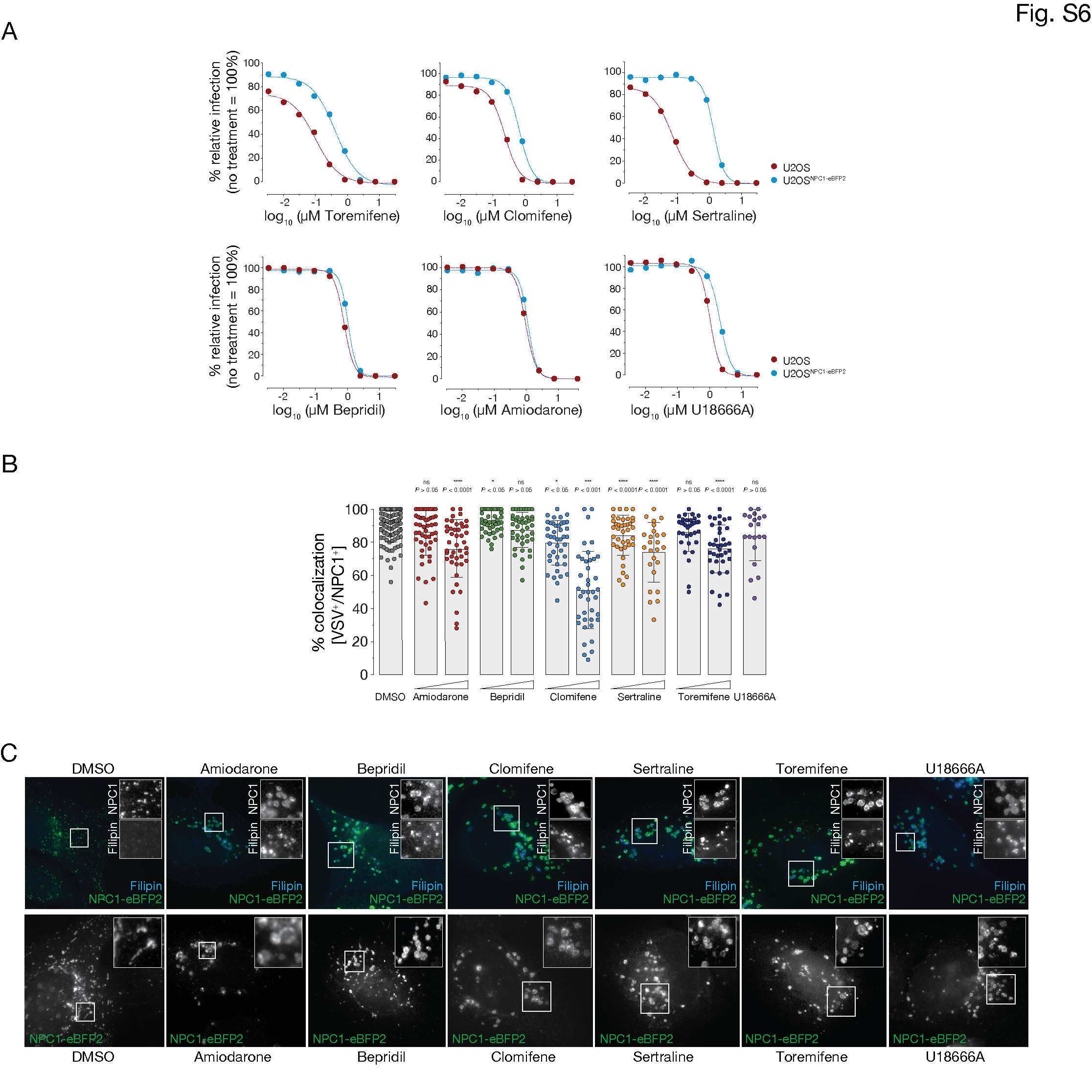

Supplement: FIG S6 [file mBio.03100-20-sf006.tif]
